# Decreased neutrophil levels in mice with traumatic brain injury after cape administration

**DOI:** 10.1016/j.amsu.2020.04.015

**Published:** 2020-05-04

**Authors:** Rizha Anshori Nasution, Andi Asadul Islam, Mochammad Hatta, Prihantono

**Affiliations:** a Department of Neurosurgery, Pelamonia Hospital, Makassar, Indonesia; b Doctoral Program of Medical Sciences, Faculty of Medicine, Hasanuddin University, Makassar, Indonesia; c Department of Neurosurgery, Faculty of Medicine, Hasanuddin University, Makassar, Indonesia; d Clinical Microbiologist Program, Faculty of Medicine, Hasanuddin University, Makassar, Indonesia; e Department of Surgery Faculty of Medicine, Hasanuddin University, Makassar, Indonesia

**Keywords:** Traumatic brain injury, Myeloperoxidase, Caffeic acid phenethyl ester, Blood brain barrier

## Abstract

**Introduction:**

Peripheral leukocytes can worsen brain damage due to the release of cytotoxic mediators that interfere the blood brain barrier function. One of the oxidants released by activation leukocyte is hypochlorite acid (HOCl) which is formed through the myeloperoxidase (MPO)-H2O2–Cl^-^ system. The neuroprotective effects of an experimental anti-inflammatory drug Caffeic Acid Phenethyl Ester (CAPE) tested in a Traumatic brain injury (TBI) model using Myeloperoxidase (MPO) analysis.

**Methods:**

This study compares the acute inflammatory response to TBI over time, as measured by MPO activity. Adult Sprague mice were treated for head trauma with *marmarou model*. At 24 h before trauma, all animals were blood test (n = 10) to examine MPO, then the animal was divided into 2 groups of injured animals treated with CAPE (n = 5), and those not treated with CAPE (n = 5). We used the MPO test to identify the level of polymorphonuclear leukocytes (PMNL) on day 4 and day 7.

**Results:**

Showed an increase in PMNL infiltration in CAPE untreated animals, this change significantly (P < 0.05) decreased at group of animals treated with CAPE. MPO serum activity in the CAPE untreated group vs treated with CAPE on day 4 ± 11920410.076 (Standard deviation {SD} 895355.169) vs 6663184.485 (SD 895355.169) p < 0,05 and on day 7 ± 14223286.992 (SD 802762.401) vs 9284222.028 (SD 953098.093) p < 0,05. These data indicate that MPO activity after TBI increases on day 4 also on day 7 and improves after being treated with CAPE.

**Conclusion:**

CAPE can reduce Neutrophil serum levels there by preventing brain damage in TBI.

## Highlights

• Peripheral leukocytes can worsen brain damage due to the release of cytotoxic mediators that interfere the blood brain barrier function.
• One of the oxidants released by activation leukocyte is HOCl which is formed through the MPO-H2O2–Cl-system.
• CAPE administration in mice with traumatic brain injury can inhibit the formation of myeloperoxidase as a marker of accumulated neutrophils.
• CAPE can reduce Neutrophil serum levels there by preventing brain damage in TBI.

## 1 Introduction

Inflammatory processes play a key role in secondary brain injury after trauma, specifically contributing to changes in cerebral blood flow, edema, intracranial hypertension, and ultimately nerve death [1]. In inflammatory conditions, the blood brain barrier (BBB) function can worsen neuronal dysfunction [2]. Some issues related to BBB dysfunction include kinin system, excitotoxicity, recruitment/activation of neutrophils, mitochondrial dysfunction, Nicotinamide adenine dinucleotide phosphate (NADPH) oxidase or nitric oxide synthase activation, and macrophage/microglial activation, all of which converge in reactive species formation [[1], [2], [3]].

MPO is a reliable enzyme marker for PMNL and is found in azurophilic granules, one of many enzymes secreted by inflammatory cells including neutrophils, macrophages and microglia, which can be used as polymorphonuclear activity marker [4]. Increased levels of MPO can cause endothelial dysfunction, upregulate the synthesis of nitric oxide and damage the function of active lipoproteins. In addition, MPO is also involved in the blood brain barrier damage at bacterial meningitis [5,6].

Neutrophils play important role in the inflammatory response outside the central nervous system (CNS), are thought to participate in the inflammatory response after brain injury. MPO testing has been shown to be a reproducible and objective method for measuring PMNL accumulation in myocardial infarction, skin inflammation, lung infections, and inflammation of the intestine [7,8]. This test was developed by Bradley and colleagues (1982) to measure the accumulation of neutrophils in inflammatory skin lesions and was modified by Barone and colleagues (1991) with MPO testing used in brain tissue [9]. Since then, this technique has been used to measure neutrophils accumulation of the brain in the regulation of ischemic and traumatic brain injury [10,11].

CAPE is one of the most effective lipophilic antioxidants [12,13]. CAPE has been shown to use the protective effect *in vivo* against hypoxia death cell in ischemic injury of neonatal brain trials also stabilize hemodynamics and areas at risk of reperfusion in ischemic heart (IR) [14,15]. CAPE has been shown to increase NO levels in plasma and in the brain, reducing brain infarction and cerebral vasospasm in mice [16]. However, the molecular mechanisms underlying CAPE mediating increasing NO levels and reducing infarction in the brain have never been explained before. CAPE's anti-inflammatory action is further documented through reduced ED1 expression (a marker of activation macrophage/microglia). This treatment inhibits apoptotic cell death by decreasing caspase 3 regulation and increasing regulation of the anti-apoptotic B-cell lymphoma-extra large (Bcl-xL) protein [14,15,17].

This study investigates the acute inflammatory response to traumatic brain injury by evaluating the time course of neutrophil accumulation in the brain, as measured by MPO activity, and comparing the responses between groups treated with CAPE and those not treated by CAPE.

## 2 Materials and methods

### 2.1 Surgical procedure

All animal procedures were conducted and approved by the Ethics Commission Faculty of Medicine, Hasanuddin University Number: 771/UN4.6.4.5.31/PP36/2019. All surgical procedures are carried out using aseptic techniques. Ten male Sprague-Dawley mice (over 2 months, weighing 280–300 g) had free access to food and water until the time of the study. Mice were placed in two groups: (1) CAPE (+) head trauma, (2) CAPE (−) head trauma.

Anesthesia was induced with diluted ketamine at a dose of 100 mg/kg, performed head trauma treatment with amarmarou model modified [18]. Surgical procedure begins with aseptic procedure using Povidone Iodine, carried out coronal incision through the midline of the brain, then boorhole is done with highspeed drill strength slowly until the exposure of the dura mater as a gap for Knabel Tang entry is done craniectomy with a diameter of 1.5 cm, 20 gr mass was dropped from a height of 20 cm by passing through the tube as a medium of mass fall, the head of a mice that has been exposed to the dura is placed just below the tube, the mass of 20 g [19] hit exactly the mice head that has been exposed to the dura, to ensure the trauma model has caused damage to the brain, one hits mice *sacrificed* to do a pathology examination with hematoxylin staining in the presence of brain tissue bleeding, the other mice were treated according to craniotomy procedures, suturing the wound iris after it was smeared with antibiotics zalf. All procedures are surgical performed aseptically by adhering to the principle of sterility. After craniotomy, all trauma models’ animals were treated at room temperature for recovery and returned to their cages.

### 2.2 CAPE administration

CAPE was purchased from Sigma-Aldrich Pte.ltd with Reagan Number 10454-70-9 prepared in saline solution and given by intraperitoneal injection (IP) following right after 30 min after trauma at a dose of 10 mg/kg, then repeated every day for 7 days, while the control group was given a placebo as well as the CAPE.

### 2.3 Sample examination

Blood taken 24 h before the treatment which is used as the base value of each sample, then blood is taken on the 4th day and 7th day after the treatment. All blood samples were examined by MPO Sandwich ELISA with Catalog No. Ls-F24875 purchased from Life Span Bio Sciences, Inc.

### 2.4 Statistical analysis

All data are presented as mean ± SD. Data were processed and analyzed using SPSS for Windows 23 software (IBM Corp. Released 2015. IBM SPSS Statistics for Windows, Version 23.0. Armonk, NY: IBM Corp.). MPO levels were tested with *Independence T test.* P values less than 0.05 were considered statistically significant.

## 3 Results

All mice survived after trauma until an experimental time was predetermined reached.

### 3.1 Characteristics of research subjects

Characteristics of the animal models used, such as body weight, are listed in the following table (Table 1):

**Table 1:** *Sprague* dawley mice body weight data.

	Mice Body Weight (gram)	p-Value
**Mean**	**290.07**	**0.155**
**SD**	± **10.48**	

Using the *lavene's homogeneity test*, it shown that the p-value >0.05, so it can be stated that body weight of mice did not significantly different.

**Fig. 1:**
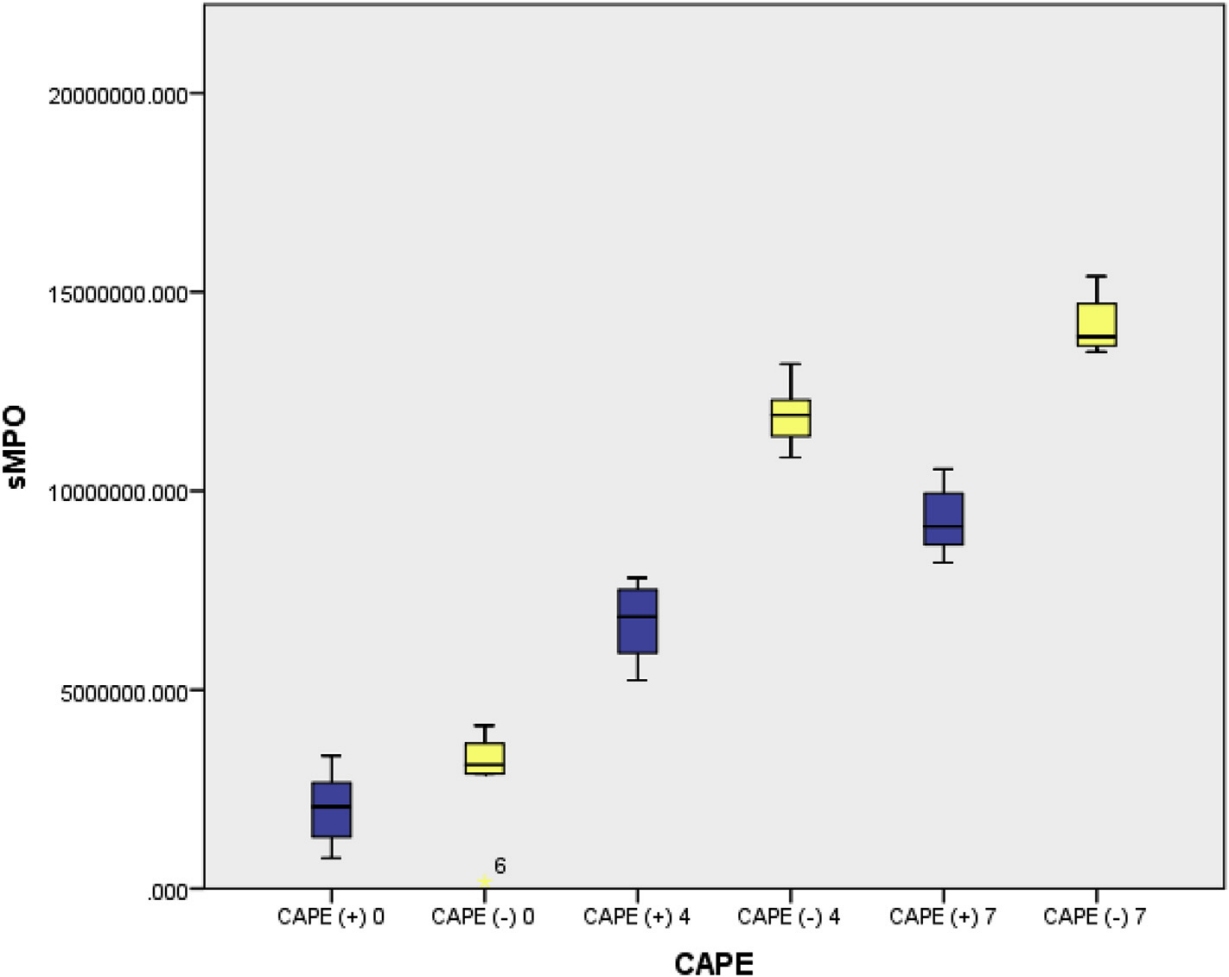
Boxplot Graph of Daily AQP-4 serum in mice with and without CAPE Administration.

Average MPO levels in mice with traumatic brain injury on days 4 and 7, were found to be lower with CAPE administration than without CAPE and there was a significant difference in MPO levels in mice that were given CAPE compare to mice without CAPE (p < 0.05). On day 7, MPO levels both with and without CAPE administration were higher than on day 4 (Table 2). This can also be seen clearly from the Boxplot chart below (Fig. 1):

**Table 2:** MPO levels in Sprague Dawley Mice with brain injury.

Day	CAPE Administration	MPO		Mean Difference	*P*
		Mean	SD		
0	(+)	2027129.639	1031180.882	−758375.422	0.386
	(−)	2785505.062	1537290.979		
4	(+)	6663184.485	1078509.605	−5257225.59	0.00
	(−)	11920410.076	895355.169		
7	(+)	9284222.028	953098.093	−4939064.96	0.00
	(−)	14223286.992	802762.401

## 4 Discussion

Leukocyte activation in the immune response is a key event that triggers regulation in the release of enzymes from intracellular granules of superoxide, through assembly the NADPH-complex oxidase in the plasma membrane. MPO is released extracellularly and into phagosome compartments by neutrophils, monocytes, and some tissue macrophages. In addition to the processes, peripheral leukocytes can worsen brain damage due to the release of cytotoxic mediators that interfere with the blood brain barrier function. One of the oxidants released by leukocyte activation is HOCl which is formed through the MPO-H2O2–Cl^-^ system [7,20].

Neutrophils accumulation in the brain, as measured by MPO activity, also increases at 24 and 48 h and improves in day 7 [9]. This increase in MPO activity also occurs after 24 and 48 h post traumatic brain injury [7]. This study examined MPO levels in the serum of mice treated with traumatic brain injury. CAPE Administration to mice with traumatic brain injury showed a significant decrease in MPO levels. The BBB structure separates brain tissue from blood flow selectively [21]. The most important structure of BBB is endothelial cells capillary which are connected to one another by *tight junctions* [2]. The opening of the *tight junction* is the main key to vasogenic edema [22]. Extracellular proteins such as occludin, claudin, and junctional adhesion molecules are the molecular composition of *tight junctions* [23,24]*.* [24,25] If this extracellular protein is excess, it will be released through phagocytic mechanism by astrocytes and microglia [23,25,26].

Post-injury CAPE administration effectively reduces BBB permeability, although its mechanism is not yet known with certainty, it is associated with an increase in claudin protein binding 5 [17]. Research shows that CAPE is oxidized by active neutrophils, and it is not a coincidence that myeloperoxidase is a protein that is most abundant in neutrophils, which are the main defense against pathogens and main effector cells in various inflammatory responses, so the involvement of CAPE in the metabolism of MPO is very clear [5,7,25]. The results of this study show a significant decrease of MPO levels in blood serum after traumatic brain injury that receives CAPE. This proves that CAPE can react directly to MPO thereby inhibiting the inflammatory process of the blood brain barrier which can worsen neuronal dysfunction. In addition to inhibiting MPO, CAPE can also reduce MPO activity in blood vessels by two mechanisms. First, CAPE has a potent anti-oxidant effect against HOCl produced by the MPO-H2O2–Cl^-^ system [7]. Second, the anti-inflammatory properties of CAPE can reduce the infiltration of blood vessel walls by MPO secreted by immunocompetent cells.

## 5 Conclusion

CAPE administration in mice with traumatic brain injury can inhibit the formation of myeloperoxidase as a marker of accumulated neutrophils, that accumulate after traumatic brain injury thereby preventing brain damage in traumatic brain injury.

## Provenance and peer review

Not commissioned, externally peer reviewed.
